# Reducing consumer materialism and compulsive buying through emotional intelligence training amongst Lithuanian students

**DOI:** 10.3389/fpsyg.2022.932395

**Published:** 2022-10-18

**Authors:** Rosita Lekavičienė, Dalia Antinienė, Shahrokh Nikou, Aušra Rūtelionė, Beata Šeinauskienė, Eglė Vaičiukynaitė

**Affiliations:** ^1^Faculty of Social Sciences, Arts and Humanities, Kaunas University of Technology, Kaunas, Lithuania; ^2^Faculty of Public Health, Lithuanian University of Health Sciences, Kaunas, Lithuania; ^3^Faculty of Social Sciences, Business and Economics, Åbo Akademi University, Turku, Finland; ^4^Department of Computer and Systems Sciences, Stockholm University, Kista, Sweden; ^5^School of Economics and Business, Kaunas University of Technology, Kaunas, Lithuania

**Keywords:** emotional intelligence, training, consumer materialism, compulsive buying, experiment

## Abstract

Consumers’ inclinations towards materialism and compulsive buying are influenced by a variety of factors. Materialistic consumers face maladies that cause stress and lower subjective well-being and are unable to control their buying behaviour that in turn leads to social and financial issues. This paper aims to investigate the effect of emotional intelligence training on consumers’ materialism and compulsive buying. The experimental design involves 36 respondents across both groups. Findings confirm the hypothesis that ability-based training programmes can help consumers improve their emotional intelligence whilst also lowering their levels of materialism and compulsive buying. In sum, the results extend the existing literature on consumer materialism by providing an explanation on how specific emotional ability-based training can diminish materialistic and excessive buying inclinations. The development of emotional intelligence skills-based training programmes contributes to more sustainable consumer behaviour, mitigating the vulnerability to materialism and related addictive behavioural consequences.

## Introduction

The issue of consumer materialism and compulsive buying has received increasing attention from specialists of various fields, such as marketing, psychology, or health sciences. Studies show that one in 20 consumers experience addictive buying behaviour driven by a propensity for materialism (Maraz et al., 2016). The consequences of compulsive buying include various social and psychological ills that affect not only the person exhibiting such behaviour (e.g., lower wellbeing, more frequent interpersonal conflicts due to uncontrolled behaviour, financial problems, etc.) but also the world in general: excessive consumption leads to more landfills, higher energy use and carbon emissions into the atmosphere (e.g., Kellett and Bolton, 2009; Otero-López and Pol, 2013; Müller et al., 2015; Omar et al., 2021; Rodrigues et al., 2021 and others). The problem of irrational consumption, hoarding and compulsive buying behaviour has been especially highlighted by the COVID-19 pandemic. Recent research shows that anxiety in the face of the threat of death increases the propensity for materialism and hoarding (Song et al., 2020; Usher et al., 2020).

This background indicates the continuing relevance of the problem of consumer materialism and compulsive buying. Following this, a question arises: are there ways to influence an individual’s thinking, attitudes, and behaviour to reduce materialistic tendencies and, at the same time, excessive buying. Some research suggests that personalities with higher emotional intelligence are less likely to exhibit materialistic tendencies, and less likely to succumb to compulsive buying behaviour (Lin and Chuang, 2005; Rose and Segrist, 2012; Nair and Das, 2015; Tariq et al., 2021). Emotional intelligence is defined as a set of abilities that help to cope with various emotional information (Mayer and Salovey, 1997) and, in relation to impulsive purchases, to help an individual cope with emerging negative emotions and feelings and choose a more thoughtful course of action. It should be noted that previous studies hypothesise and investigate the relationship between emotional intelligence and materialism and compulsive buying, research has generally been conducted using cross-sectional research design (e.g., Maddi et al., 2013; Park and Dhandra, 2017; Rūtelionė et al., 2022), which limits causal inferences. Since research supports the idea that emotional intelligence can be developed (Cherniss and Goleman, 2001; Foster et al., 2015; Hodzic et al., 2018), the aim of our study is to identify how the development of emotional intelligence affects materialism and one’s propensity for compulsive buying (emotional intelligence training-based experiment). As research of this type has not been conducted before, the present scientific study provides new knowledge towards solving the problems discussed. The paper will further analyse the already available scientific data on emotional intelligence and its development, the links between emotional intelligence and materialistic attitudes and compulsive buying, and present a study conducted with Lithuanian university students which includes experimentation and the results of various assessments (emotional intelligence, compulsive buying, materialistic attitudes, and their changes).

## Literature review and hypothesis development

### Emotional intelligence and development possibilities

Emotional intelligence is one of the most commonly used psychological terms in popular literature (e.g., Hogeveen et al., 2016). Within scientific literature this concept became established in 1990 (Mayer et al., 1990). Recently, researchers have proposed three theoretical models of emotional intelligence: ability, trait, and a mixed model (Gilar-Corbi et al., 2019). Supporters of the ability theory understand emotional intelligence as processing of emotional information. In other words, it is interpreted as the mental ability to recognise and regulate one’s own emotions as well as emotions of others (Mayer and Salovey, 1997). The model of Bar-On expands the concept of emotional intelligence developed by Mayer and Salovey (1997) by discussing personality traits that are revealed through a person’s self-reflection, influencing the ability to cope with pressure and the demands of their environment (Bar-On, 1997; Ekman et al., 2013). Literature analysis also reveals a third concept of emotional intelligence – the mixed model (Spielberger, 2004). The mixed model treats emotional intelligence as a combination of emotional skills and dimensions of a personality. However, some authors such as Nelis et al. (2009) argued that such conceptualisation of emotional intelligence can be divided into three aspects: knowledge, ability, and traits.

The existence of different emotional intelligence models determines the ongoing discussion amongst researchers on the definition and validity of this phenomenon; however, it is clear that emotional intelligence includes a number of emotional abilities, such as recognition of one’s own emotions and emotions of other people, understanding how emotions affect the behaviour of a person, and the ability to regulate one’s own emotions and emotions of others, as well as the ability to respond effectively, and to behave (e.g., adapt, control stress, develop interpersonal relations, and maintain a good mood) accordingly (Pérez-Fuentes et al., 2018). One of the pioneers of emotional intelligence research defines it as “a whole made of interrelated emotional and social competencies, skills and facilitators that determine how effectively we understand and express ourselves, understand others and relate to them, and cope with daily demands” (Bar-On, 2005, p. 3).

Despite the ongoing disagreements on the definition of emotional intelligence, its operationalisation, advantages and shortages of models, it is agreed that individuals with higher emotional intelligence are more capable to develop interpersonal relations, experience a state of higher level of psychological well-being, enjoy better mental health and distinguish themselves with academic and work achievements (Ryan and Deci, 2001; Furnham, 2009; Kee et al., 2009; Zeidner et al., 2009; Sánchez-Álvarez et al., 2016). As a result, it is reasonable to argue that high emotional intelligence is an important psychological characteristic that confers numerous advantages. Thus, substantiated questions arise whether emotional intelligence can be purposefully developed and whether development programmes can indeed substantially improve it. It is important to note that studies on the effectiveness of emotional intelligence training have not been conclusive. Even though the majority of studies show that EI training improves emotional competence (Jdaitawi et al., 2011; Hodzic et al., 2018; Kotsou et al., 2018), some studies provide no evidence. For example, Orak et al. (2016) found that an EI educational programme had no effect on the EI skills of first-year nursing students.

As we have discussed, the notion of emotional intelligence proposed by different authors of theoretical concepts differs, but these concepts also share some overlapping aspects, one of which is an attitude towards the possibilities of development of emotional intelligence. Most researchers of emotional intelligence admit that it can be developed and that development programmes can be effective (Brackett et al., 2010; Harrison and Fopma-Loy, 2010; Foster et al., 2015; Hodzic et al., 2018). However, the theoretical approach of development programmes is very important. According to the ability theory, emotional intelligence has more potential to change aspects of a personality when compared to the trait model. It has been observed by Mayer Salovey that emotional intelligence is related to the ability to learn emotional skills (Ackley, 2016 cit. Mayer and Salovey). The position that emotional intelligence can be developed is also supported by other authors, those who stick to the trait and mixed approach, (e.g., Cherniss and Goleman, 2001; Bar-On, 2005). However, research shows that programmes based on the ability approach are more effective than the mentioned trait and mixed approaches (Hodzic et al., 2018).

Recently several interventional programmes of emotional intelligence have been developed and implemented (Hodzic et al., 2018). The content and methods of EI development depend on the objective of the interventional programmes, population for which the specific programme is dedicated, and the theoretical approach used by developers of appropriate programme. For example, some programmes are oriented towards individual aspects of emotional intelligence, e.g., regulation of emotions (Shaffer et al., 2019), or emotional awareness (Uzun and Lok, 2022). However, it is assumed that programmes encompassing wider indicators of emotional intelligence are more effective (Hodzic et al., 2018). To develop emotional awareness, role play is often used to: (i) understand emotions of others – facial recognition (in photographs), and (ii) improve expression of emotions – techniques for verbal and non-verbal skill development, etc. Some emotional intelligence programmes are performed face-to-face; however, online or hybrid programmes are becoming more and more popular. Online execution of interventions provides a number of advantages – e.g. it allows saving time and finances of participants of the programmes whilst the quality is not inferior to the contact of the programmes (Kemper et al., 2015).

Programmes dedicated to the development of emotional intelligence significantly differ based on their theoretical and methodological validity, objectives, duration, context in which they are applied, and the like, therefore, comparison of programmes is complicated. Justification for the development of emotional intelligence is also impeded by the fact that some development programmes are not based on evidence, i.e., their effectiveness has not been verified, therefore, results of such programmes cannot be trusted. Researchers emphasise that even though emotional intelligence development programmes are promising, proof of their effectiveness and usefulness is mandatory (Schutte et al., 2013), making relevant, reliable, and accurate assessment instruments necessary for the substantiation of these programmes (Zeidner et al., 2002). Overall, emotional intelligence training is found to positively affect adults’ emotional intelligence, as shown in the study of Mattingly and Kraiger (2019).

It is important to note that whilst trying to assess the effectiveness of emotional intelligence development, it has been determined that development programmes bring about positive changes not just for emotional intelligence, but also for other psychological constructs (Mikolajczak et al., 2009), e.g., satisfaction with life, self-appreciation, self-efficacy, psychological empowerment, and the like (Karimi et al., 2020). For example, students who took part in an emotional intelligence mentoring programme showed an increase in gratitude (Bryant and Aytes, 2021). Furthermore, programmes based on emotional intelligence abilities have been shown to improve mindfulness of food choices, demonstrating that emotionally trained consumers can gain control over their food choices (Kidwell et al., 2015). In other words, teaching emotional intelligence has a positive effect on diverse and important spheres of life (Schutte et al., 2013).

Emotional intelligence can also be developed to overcome some risk factors or difficulties. It can be considered one of the most important safety measures that guarantees one’s mental resistance (Extermera, 2020) and good mental health (Persich et al., 2021). For example, Slaski and Cartwright (2003) found that a developmental EI training programme not only increased emotional intelligence but also had a significant and positive effect on health and well-being measures. Similarly, Nelis et al. (2011) demonstrated that emotional intelligence could be improved even with relatively brief training, demonstrating the positive implications for psychological well-being and being sufficient to decrease neuroticism. Ruiz-Aranda et al. (2012) found that the emotional intelligence educational programme designed to help adolescents develop skills in perceiving, facilitating, understanding, and managing emotions was effective in promoting mental health. Development of emotional intelligence could be applied in various areas. As Kotsou et al. (2018) suggest, EI interventions could be a promising tool even in clinical settings, such as treating work burnout, chronic stress, and alcoholism-related problems. Correlational studies show that people with high emotional intelligence have fewer symptoms of anhedonia and are less prone to suicidal ideation (Abdollahi et al., 2020). Therefore, it can be suggested that development of emotional intelligence may function as a safety measure that decreases materialism and compulsive buying.

### Materialism and emotional intelligence

Materialism is a psychological construct that reflects how important acquiring wealth, property, status, and image are to an individual compared to other goals in life (Kasser, 2016, 2018). It is also described as acquisition and use of material wealth to pursue one’s goals in life or certain states of being (Richins, 2004). In other words, materialism is a value system oriented towards material wealth whilst perceiving it as a major indicator of an individual’s success and a means to attain happiness. Acquired and accumulated things produce a feeling of happiness to an individual because they bring them nearer to the conceived ideal self-concept and the desired standard of living.

Materialism is conceptually approached in two ways: on the one hand, it is seen as having a positive side for its ability to increase motivation to work and contribution to the growth of the economy by stimulating the demand for goods (Kilbourne and LaForge, 2010; Sirgy et al., 2013). On the other hand, within scientific literature, the perception of materialism as possessing a dark side due to consumer behaviour prevails (Mick, 1996). Studies have long ago shown negative consequences of materialism to various aspects of life (Jiang et al., 2021), e.g., materialism is linked to an increased level of anxiety and depression, lower satisfaction with life, poorer physical health, and lower self-esteem (Kasser and Ryan, 1993; Chaplin and John, 2007; Otero-López et al., 2011; Jiang et al., 2021). Materialism is also linked to negative emotionality whilst buying relates with the desire to get rid of negative feelings and to improve one’s mood (Donnelly et al., 2013).

Materialistic behaviour can be explained by a person’s dissatisfaction with oneself and their standard of living (Donnelly et al., 2016). For example, the young people raised during periods of societal insecurity and increased unmarried parenthood were more likely to support materialistic values (Twenge and Kasser, 2013). Individuals with materialistic values construct their identity according to the things that they have acquired (Carter and Gilovich, 2012; Richins and Chaplin, 2021). Usually, such individuals try to achieve certain goals by acquiring and using various objects, but after having reached the set goal they immediately come up with other, greater ones, thus setting off a constantly repeated process.

Materialism is closely connected to the emotional sphere: emotions may determine materialistic inclinations. Possession of things to materialists equals to the feeling of happiness. Many consumers expect material wealth to bring contentment, but studies show the opposite to be true – materialists are less happy (Donnelly et al., 2016), and any negatively appreciated emotion may increase materialism (Longmire et al., 2021). For example, fear is closely connected with materialistic behaviour (Ruvio et al., 2014; Longmire et al., 2021). When consumers experience fear they behave more materialistic, as objects provide them with a sense of security. Therefore, consumers reduce their fear, anxiety, and feelings of insecurity by acquiring goods and thus materialism increases (Kasser and Sheldon, 2000; Chang and Arkin, 2002; Christopher et al., 2006).

As it has been mentioned earlier, emotional intelligence is one’s ability to process their own emotions and emotional information obtained from the environment (Mayer et al., 2008). Researchers of emotional intelligence, depending on which specific theoretical model they represent, distinguish different indicators for emotional intelligence, but two indicators are most obviously related to materialism – emotional awareness and regulation of emotions.

It is believed that emotional awareness is an essential aspect of emotional intelligence (Hogeveen et al., 2016). Understanding of emotions consists of the ability to perceive connections between diverse emotions and to notice how emotions change in the long run and under different situations (Rivers et al., 2007). Understanding of one’s own emotions comprises not only the distinction of the experienced emotion by its type, but also the ability to identify the intensity and duration of the emotion (Frijda, 2007), as well as the capacity to exactly understand the influence of one’s own emotions on behaviour (Bagshaw, 2000). Therefore, understanding emotions may serve as a barrier to materialistic behaviour.

Precise understanding of one’s own emotions is related to the other indicator of emotional intelligence – regulation of emotions (Barrett et al., 2001; Estévez et al., 2020). Regulation of emotions, just as perception, is considered to be an important psychological skill (Robazza et al., 2022). Effective regulation of emotions creates the basis for one’s adequate adaptation, whilst inability to regulate is understood as difficulty to control impulses (Strack and Deutsch, 2004). Regulation of emotions may be defined as a purposeful process aimed to affect the type, duration, and intensity of experienced emotions (Peña-Sarrionandia et al., 2015). Regulation includes efforts to reduce negative or reinforce positive emotional experiences, expression of emotions (Gross and Thompson, 2007; Gross, 2010) and attempts to adequately use emotions in specific situations (Papadogiannis et al., 2009). Individuals with a higher indicator of emotional intelligence do not let their emotions automatically affect their behaviour, therefore, it is likely that people who regulate their emotions better can act as more rational consumers and are better at controlling tendencies of materialistic behaviour.

It should be noted that emotional intelligence, which also includes understanding, managing, and harnessing other people’s emotions, has been positively linked to subjective well-being (Schutte and Malouff, 2011). In addition, materialism is well documented in the literature as being negatively associated with subjective well-being. Furthermore, some authors show that individuals with alexithymia, characterised by a reduced capacity for empathy, have significantly higher materialistic values than those without alexithymia (Vredeveld, 2021). Moreover, studies show a negative relationship between alexithymia, and the perceived quality of intimate relationships (Humphreys et al., 2009). In a related study, Zhao et al. (2022) found that alexithymia is positively connected with loneliness, and internet addiction. Similarly, there is empirical evidence that loneliness promotes materialism (Pieters, 2013). Thus, the literature supports the assumption that abilities to understand and manage the emotions of others should also be held accountable for the development of materialism.

### Compulsive buying and emotional intelligence

Compulsive buying can be defined as a casual, irresistible, spontaneous, and uncontrollable urge that leads an individual to repetitive purchasing, which may cause many negative consequences such as financial, social, and personal problems (e.g., Dittmar et al., 2007; Kellett and Bolton, 2009; Brougham et al., 2011; Otero-López and Pol, 2013; Müller et al., 2015; Omar et al., 2021; Rodrigues et al., 2021). It is also important to note that problems caused by excessive purchases (falling self-esteem, indebtedness, declining relationships with loved ones, etc.) do not affect the individual in any way and they continue to spin in a vicious circle without being able to control their behaviour (Claes et al., 2010; Müller et al., 2015).

Prior studies analyse the origins of compulsive buying. For example, according to Rodrigues et al. (2021), compulsive buying behaviour does not depend only on a single variable, but rather on a combination of emotional, genetic, psychological, social, and other factors. Particularly many associations are found between materialistic attitudes of an individual and compulsive consumption. For example, research by Roberts et al. (2008), reveal the role of parents in the formation of adolescent materialism and compulsive buying, and later has been confirmed by further studies such as research by Islam et al. (2018), and Tarka (2020), who explore the relationship between materialism, and compulsive buying amongst adolescents and the young adults. Moreover, Mowen and Spears (1999), Reeves et al. (2012), and Villardefrancos and Otero-López (2016) suggest that materialistic individuals are more prone to compulsive buying due to low self-esteem. In addition, Garđarsdóttir et al. (2009) emphasise that the subjective well-being of consumers who exhibit such behaviour is particularly low, so they try to experience and gain happiness by purchasing material things. Furthermore, Kasser and Kanner (2004) found that individuals experiencing insecurity due to peer rejection and social exclusion are more dependent on compulsive consumption, and abundant buying may serve as an endeavour to move closer to the ideal self (Dittmar, 2005). Rustagi and Shrum (2017) argue that compulsive buying is one of the main negative consequences of materialism, and study by Harnish et al. (2019) also confirms that materialism is associated with and predictive of compulsive purchasing.

As such, one can ask: *what is the relationship between the level of emotional intelligence and compulsive buying?* There are relatively few studies of this nature, but literature provides a theoretical basis for hypothesising this relationship. For example, some authors argue that poor emotional self-regulation is the basis for compulsive buying (e.g., Rose and Segrist, 2012; Jung, 2017; Tariq et al., 2021). Moreover, Saraneva and Sääksjärvi (2008) found that the emotions of compulsive buyers are much more complex, and an emotional continuum rises and falls during buying. Maddi et al. (2013); for example, discovered that EI does not protect against compulsive buying. The complex emotions of such consumers are discussed by other authors indicating that compulsive buying is accompanied by a subsequent feeling of relief along with guilt arising from addictive behaviour (e.g., Valence et al., 1988; D'Astous et al., 1989; Weinstein et al., 2016; Gallagher et al., 2017; Müller et al., 2019). Furthermore, Kun and Demetrovics (2010) emphasise the importance of two emotional intelligence components that play a central role in addictions: (i) decoding and differentiation of emotions, and (ii) regulation of emotions. These studies echoe research by Lin and Chuang (2005), who found that there is a negative association between adolescents’ emotional intelligence and propensity to impulsive purchasing, i.e., individuals with lower emotional intelligence are more dependent on purchasing. This is also confirmed by Nair and Das (2015), as consumers with a higher level of emotional intelligence are less inclined to engage in impulsive buying. Recent research also reflects this trend as when consumers have a high level of emotional intelligence, their involvement in impulsive buying is low, and they are more satisfied with their lives (Tariq et al., 2021), as well as good emotion management skills protect consumers from unplanned purchases, enabling them to make better purchasing decisions (Khan and Khan, 2021).

Thus, it can be speculated that an emotional intelligence development programme focused explicitly on raising an individual’s level of emotional competence would have an impact on compulsive behaviour (CB). Accordingly, four main elements (skill groups) of the emotional intelligence programme should be emphasised: (1) the ability to fully and accurately perceive, comment on and recognise emotions, (2) the ability to evoke appropriate emotions for the current situation and to have emotion control that helps to understand one’s own and others’ emotions, (3) the ability to read information received from emotions, and (4) the ability to regulate emotions manifesting as impulse force (Weisinger, 1998; Campo et al., 2015; Hodzic et al., 2018; Kotsou et al., 2018; Mattingly and Kraiger, 2019). All four key elements of emotional intelligence development listed above are necessary to neutralise compulsivity. Emotions strongly affect an individual’s thinking and behaviour, i.e., emotions can change and disrupt cognitive structures. This ability of emotions to change the cognitive system can lead to different results, and unrecognised and uncontrolled positive and negative emotions can steer thinking in both negative and positive directions. During emotional intelligence training, individuals learn to become acquainted with the experienced emotions, and to identify their meaning, the periods, and circumstances in which they recur, and so on (Yılmaz, 2012). It is these aspects that are very important in correcting compulsive behaviour, hence the following hypotheses are formulated:

*Proposition 1:* Consumers who complete an ability-based training programme will have higher levels of emotional intelligence than those in a control group.

*Proposition 2:* Consumers trained in emotional abilities will exhibit significantly lower levels of materialism and compulsive buying than those in a control group.

In summary, it should be noted that the authors acknowledge that materialism is about one’s belief and compulsive buying is about one’s emotional impulse. Therefore, it is plausible to assume that different types of intervention may be required to change materialistic buying or compulsive buying. To address this issue, we argue that materialism and compulsive buying are distinct constructs, and whilst the manifestations of both constructs differ, they share the same antecedents. Both constructs are psychologically motivated in their pursuit of mood-repair (Dittmar, 2005). According to literature, both MAT and CB tendencies share similar pre-purchase characteristics, such as negative feelings as well as aversive emotional states (Donnelly et al., 2013, 2016). Consumers are motivated to cope with their self-ascribed deficiencies through escape or another coping mechanism when they are over-concerned with their negative feelings and an unpleasant self (Donnelly et al., 2016). Consumers gravitate towards materialistic endeavours because they believe in the power of material objects to transform their self-identity and bring happiness (Richins, 2017), or they tend to attach to material objects to avoid loneliness (Pieters, 2013). The same applies to CB; for example, detachment is found as a distinguishing feature of CBs (Duroy et al., 2018). In contrast to impulse buying, CB is primarily motivated by negative affect and feelings in search of relief (Darrat et al., 2016). Furthermore, there is a strong positive relationship between MAT and CB (Dittmar et al., 2007). Materialistic value enforcement is found as the strong predictor of CB (Dittmar, 2005). To sum up, literature provides support that both phenomena are driven by an abundance of negative feelings and aversive emotional states.

## Materials and methods

### Participants and sampling procedure

In this research, an experimental study was conducted amongst students from Lithuanian Universities. The research was conducted completely online due to pandemic conditions. The experimental design has been adopted with the independent variable or cause being emotional intelligence and the dependent variable or effect being materialism and compulsive buying. The causal effect of an intervention (i.e., EI training) on consumers’ emotional intelligence, materialism and compulsive buying have been evaluated on a target population (i.e., students of universities).

As many as 67 students volunteered to take part in the experiment online. Participants of the study were recruited *via* invitations placed in universities’ social media. The sample size has been calculated based on several methods. Firstly, we follow the rule that the most acceptable way of sample size determination is 10:1 ratio (i.e., 10 samples for one variable; Hair et al., 2010). In addition, we have followed the Central Limit Theorem, which declares the minimum sample size for experimental studies should be at least 30 (Matz and Hause, 2008). The students were asked to complete the initial screening questionnaire to determine their inclinations to materialism and compulsive buying (67 questionnaires were filled out). Students who participated in the initial survey questionnaire (pre-test) scored on average (*N* = 67, *M* = 2.22, SD = 1.08) for compulsive buying, and (*N* = 67, *M* = 3.04, SD = 1.23) for materialism. The respondents’ age ranged from 18 to 32, with the average age of 22.86 (SD = 3.22).

After analysing the initial data collected from the survey to be used for the experimental research, we have selected only those participants who scored above the average (i.e., *M* = 3.04) on materialism scale. Those who did not meet this criterion, were not invited to participate in the emotional intelligence training-based experiment. Thus, after excluding the participants who were not eligible to participate, the final sample consisted of 36 participants. In the next step, the participants were randomly divided into two groups: intervention (treatment) and control (received no treatment), and all variables apart from the treatment have been kept constant. Participants of the intervention (treatment) group 1 (*n* = 18) have participated in an emotional intelligence development programme. Group 2 (control; *n* = 18) did not participate in the training programme, but they were informed and encouraged to participate in the survey after the training and the experiment. The comparability of both groups is very important for experimental research; thus, we have checked the difference between the experiment and control group and found that these two groups did not differ from each other with respect to their age. In addition, the two groups were compared based on the scores respondents provided on CB (group 1, *M* = 2.36, group 2, *M* = 2.35) during the pretesting of the questionnaire. The results of independent samples test show that the groups did not differ based on the average scores (*t* (34) = 0.059, *p* = 0.718, Cohen’s *d* = 0.66) given to CB. As such, we determined that there is no difference between the groups before the experiment and training programme. The same test was applied on EI, and the results of independent samples test show that the two groups did not different in this variable before the after the experiment (*t* (34) = 0.913, *p* = 0.500, Cohen’s *d* = 0.27). It should be noted that, even though we assumed the existence of the null hypothesis, which refers to the situation that the experimental group and control group are indifferent at the initial stage and before the training (treatment); however, one could also assume that there might be a group difference based on some other variables (such as income or social class), which were not taken into account in this study.

### Instrumentation

The initial screening and pre-test questionnaire used for pre-screening was the same as the one used when measuring outcomes (post-test). Thus, pre-and post-assessment was applied to the participants aiming to evaluate the effectiveness of the EI training programme as an intervention in reducing the degree of materialism and CB. Participants from both groups completed questionnaires with the online supervision of experimenters before and after the training programme was conducted.

To measure consumer materialism, we used Richins’ (2004) 5-point Likert-type scales, (one being “strongly disagree” and five being “strongly agree”) consisting of three dimensions: centrality (seven items), happiness (five items), and success (six items). For example, we asked respondents to indicate their agreement with the following statement “My life would be better if I owned certain things I do not have.” Compulsive buying was measured using Valence et al. (1988) items consisting of 13 items in three dimensions: tendency to spend (six items), reactive aspect (four items) and post-purchase guilt (three items) using 5-point Likert-type scale, with one being “strongly disagree” to five being “strongly agree. For example, we asked respondents to indicate their agreement with the statement “When I have money, I try to spend part or the whole of it.” The emotional intelligence scale we used is the Schutte et al. (1998) 5-point Likert-type scale which consists of four dimensions [appraisal of emotions (10 items), emotion regulation of self (nine items), emotion regulation of others (eight items) and utilisation of emotions (six items)]. For example, we asked respondents to indicate their agreement with this statement “I use good mood to help myself keep trying in the face of obstacles.” The measurement scales used in the research are presented in Annex 1.

### Intervention

The intervention designed for this study is the emotional intelligence training programme. Training based emotional intelligence interventions are acknowledged as important facilitators for behavioural change in health and psychological research (Orak et al., 2016; Bryant and Aytes, 2021). However, there have been no studies carried out in which emotional intelligence training is used as an intervention for diminishing consumer materialism and compulsive buying. The programme has been developed by psychology researchers of this study and it is based on extensive analysis of this programme’s usability for behaviour change purposes. As such, the experiment executed in this research can be assumed to be the first attempt to investigate the effectiveness of emotional intelligence development in reducing the degree of materialism and compulsive buying amongst students at Lithuanian universities.

The main objectives of the EI training programme were: (i) to develop the participants’ ability to accurately identify and analyse their own emotions and that of others at a particular moment as well as help to understand aspirations, motives, and incentives to act and effectively decode nonverbal and verbal cues, (ii) to develop the participants’ ability to use conscious information about their own and others’ emotions (especially negative), and to adjust their emotional reactions to themselves, situations, and others accordingly, and (iii) to strengthen the newly formed emotional competencies in the group by modelling various realistic situations. Thus, the EI training programme covers all components of the EI model. The design of the current intervention included discussion, examples, paired exercises, emotion diaries, readings, reflection, and feedback. Those methods helped to understand and manage one’s own and others’ emotions. Some of the tasks in the programme were linked to the practical application of acquired knowledge in real life to turn them into skills (in the form of self-assigned tasks). Also, the curriculum established objective criteria to assess the change in the level of emotional intelligence of the personality after the end of the emotional intelligence programme. The duration of the programme is also crucial for the effect of a positive change in a person’s emotional intelligence (Kotsou et al., 2011; Zijlmans et al., 2011). Due to the restrictions brought about by the global pandemic, face-to-face training was not an option at the time of experiment, and an online contact method was chosen instead. The participants in the intervention group were offered 3 days long-distance training of 2 h each in June 2021. The programme was taught by two psychologists with a specialty in EI, in addition to other co-authors. The EI training programme outline is presented in Annex 2.

## Results

As mentioned, compulsive buying was measured using a scale developed by Valence et al. (1988). We obtained an acceptable reliability for this construct (Cronbach’s *α* = 0.826). Moreover, the reliability for emotional intelligence (EI), (Cronbach’s *α* = 0.807), and for the materialism construct (Cronbach’s *α* = 0.738), was also acceptable. The results of the independent samples *t*-test showed that participants in the training-based emotional intelligence intervention group reported a significantly higher level of emotional intelligence (*N* = 18, *M* = 3.66, SD = 0.26) than participants in the control group (*N* = 18, *M* = 3.22, SD = 0.35), *t* (34) = 4.203, *p* < 0.001, Cohen’s *d* = 0.31, which indicates that the training-based emotional intelligence intervention was successful. This result supports the first proposition, where it was postulated that respondents who participate in the training-based emotional intelligence intervention will have a higher level of emotional intelligence than those in a control group. In addition, we examined the four dimensions of emotional intelligence (appraisal of emotions, emotion regulation of self, emotion regulation of others, and utilisation of emotions) separately to see if the training-based intervention was effective in all four dimensions of emotional intelligence, when the two groups (experiment vs. control) are compared. The independent samples *t*-test results showed the training-based intervention to be indeed effective in all dimensions of emotional intelligence, see Table 1.

**Table 1:** Group statistics on emotional intelligence dimensions.

EI dimensions	GROUP	*N*	Mean	SD	*t*	*df*	Sig. (2-tailed)
Emotion regulation of others	Experiment	18	3.70	0.54	3.45	34	0.001
	Control	18	2.94	0.76			
Emotion regulation of self	Experiment	18	3.82	0.53	4.29	34	0.001
	Control	18	3.42	0.45			
Appraisal of emotions	Experiment	18	3.66	0.34	6.39	34	0.001
	Control	18	2.98	0.48			
Utilisation of emotions	Experiment	18	3.54	0.46	2.13	34	0.05
	Control	18	3.25	0.44			

**Figure 1:**
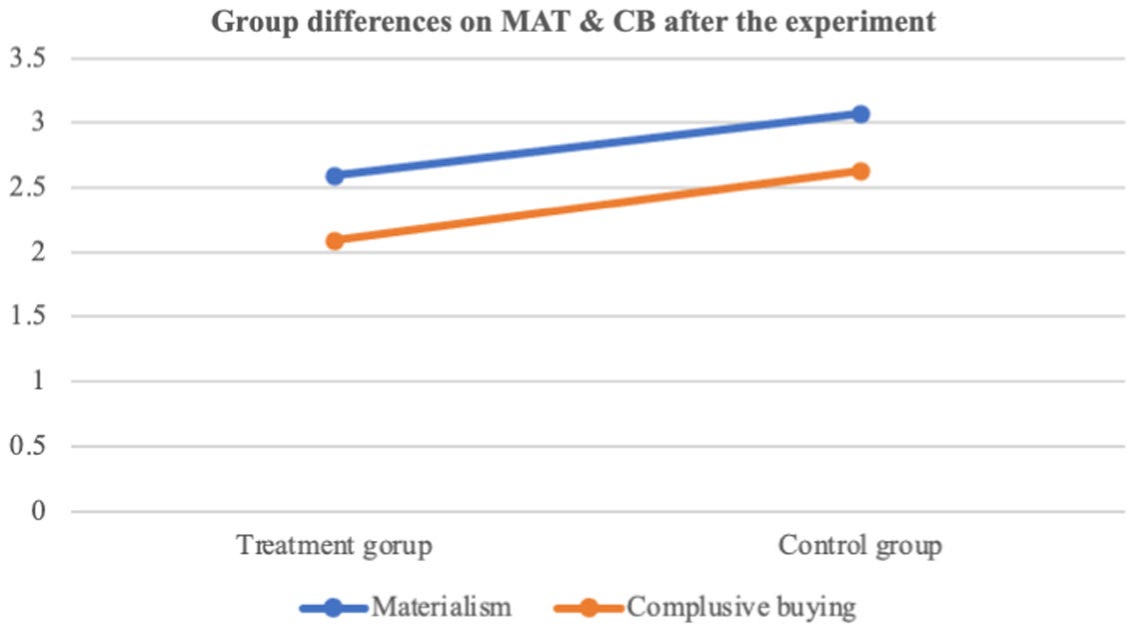
Group differences on MAT and CB after the training and experiment.

Moreover, results of the independent samples *t*-test showed that participants in the training-based EI interventions group reported significantly lower levels of materialism (*N* = 18, *M* = 2.59, SD = 0.50) than participants in the control group (*N* = 18, *M* = 3.07, SD = 0.29), *t* (34) = −3.508, *p* < 0.001, Cohen’s *d* = 0.41, as well as a lower level of compulsive buying (*N* = 18, *M* = 2.09, SD = 0.50) than participants in the control group (*N* = 18, *M* = 2.63, SD = 0.67), *t* (34) = −2.722, *p* < 0.001, Cohen’s *d* = 0.59, see Figure 1. These results indicate that the training-based emotional intelligence intervention was successful and support the second proposition, which postulates that respondents who participate in the training-based EI intervention will have lower levels of materialism and compulsive buying than those in a control group. Based on the analysis performed on the data, the results showed that emotional intelligence development training has not only helped respondents to gain a higher level of emotional intelligence, but also influenced consumers’ inclination to materialism and compulsive buying.

As we measured consumers’ materialism, which is composed of three dimensions (centrality, happiness, and success), in further analysis, we ran independent samples *t*-test to account for the impact of the training-based emotional intelligence interventions in each of these dimensions between the two groups: group 1: experimental, and group 2: control. The results show that respondents trained in emotional intelligence exhibited significantly lower levels of materialism in all three dimensions compared to participants in the control group, where no training was provided. Such that for the centrality dimension, group 1 experimental (*N* = 18, *M* = 2.74, SD = 0.69) scored lower than participants in the group 2: control (*N* = 18, *M* = 3.26, SD = 0.26), *t* (34) = −2.997, *p* < 0.005, Cohen’s *d* = 0.52. Moreover, for the happiness dimension, group 1 experimental (*N* = 18, *M* = 2.52, SD = 0.68) scored lower than participants in the group 2: control (*N* = 18, *M* = 3.39, SD = 0.39), *t* (34) = −4.708, *p* < 0.001, Cohen’s *d* = 0.55. Finally, for the success dimension, group 1 experimental (*N* = 18, *M* = 2.48, SD = 0.46) scored lower than participants in the group 2: control (*N* = 18, *M* = 3.11, SD = 0.35), *t* (34) = −4.557, *p* < 0.001, Cohen’s *d* = 0.41.

## Discussion

### Theoretical contributions

Abilities to cope with emotions is very crucial for individuals with addictions (Kun and Demetrovics, 2010). To prevent lower subjective well-being and mental health, consumers need to understand their own emotions and learn how to manage them (Extermera, 2020; Persich et al., 2021). There are many emotional intelligence, mindfulness, and other related practise-based programmes aimed at decreasing stress and psychological discomfort and assisting individuals in learning new skills that could supposedly help to tackle excessive buying problems. These programmes have been shown to improve overall health and well-being (Slaski and Cartwright, 2003). Previous experimental research by Nelis et al. (2011) also showed that even brief emotional intelligence training programmes can produce positive outcomes such as increased psychological well-being and decreased neuroticism. However, causal design-based research on the effect of emotional intelligence development on materialism and compulsive buying is not found and the effect remains unknown.

The aim of this study was to investigate the effect of emotional intelligence development on the levels of materialism and compulsive buying exhibited by Lithuanian students. The findings reveal significant differences between the experimental and control groups before and after the intervention. In summary, the emotional intelligence development training programme has increased the emotional intelligence of experimental group participants and resulted in lower self-reported scores of materialisms and compulsive buying.

The lack of studies on the effect of emotional intelligence training on materialism and compulsive buying complicates the comparison with previous findings (Ruiz-Aranda et al., 2012; Kidwell et al., 2015; Bryant and Aytes, 2021) where emotional intelligence and stress, gratitude, mental health, and mindful eating relationships have been investigated. Nonetheless, our results are consistent with other studies that show similar regularities. For example, the pattern of findings in our study is consistent with that of Ruiz-Aranda et al. (2012) who discovered that emotional intelligence training reduced negative affect, depression, social stress, and a sense of incapacity in an adolescent sample. Based on the regularities mentioned above, it is plausible to assume a similar effect on materialism and compulsive buying, as both latter phenomena are associated with increased anxiety and depression, as well as lower life satisfaction (Kasser and Ryan, 1993; Chaplin and John, 2007; Otero-López et al., 2011). Another study by Bryant and Aytes (2021) is also in line with our research results. In their study, they investigated how the emotional intelligence mentoring programme affected students’ EI, stress as well as gratitude, and confirmed that those students who participated in the mentoring programme had higher EI and gratitude levels and a lower level of stress. The development of emotional abilities has been investigated by Kidwell et al. (2015), results of which indicated that EI could be developed, helping consumers choose food in a more mindful way. Nevertheless, none of the mentioned research has included emotional intelligence as the cause and consumer materialism and compulsive buying as the consequences in their experimental studies.

Our study contradicts another causal research-based study of Orak et al. (2016) in which emotional intelligence scores of first course students were evaluated before and after emotional intelligence education course and there were no significant differences between the two study groups in terms of emotional intelligence scores. This contradiction can be attributed to the fact that the experiment participants were nurse students who feel more stress and anxiety in their daily professional environment. It is also important to stress that neither materialism nor compulsive buying were investigated in the Orak et al. (2016) study.

The findings of our study indicate the influence of the uniquely designed emotional intelligence development programme that has been tested amongst students at several Lithuanian universities. Results of experiment confirm both hypotheses: consumers who complete an ability-based training programme will have higher levels of emotional intelligence than those in a control group (H1) and consumers trained in emotional abilities will exhibit significantly lower levels of materialism and compulsive buying than those in a control group (H2). The effect size described by Cohen’s d was medium for the construct of emotional intelligence, which indicates the importance of the emotional intelligence development as a whole. Furthermore, separate dimensions of emotional intelligence that fall under the construct (perception of emotions, managing own emotions, managing others’ emotions and utilisation of emotions) also indicate the positive effect of emotional intelligence training when comparing both experiment groups.

Although emotional intelligence has been studied primarily in psychology and psychiatry, the current study found that EI training can also demonstrate predictive power in consumer behaviour, especially when dealing with materialistic concerns. Our study confirms that emotionally intelligent individuals who have been trained to understand and regulate their emotions could more efficiently cope with the possessions and buying-related preoccupations. As a result, this new finding adds to the body of knowledge in psychology and consumer behaviour about the effect of EI training on materialistic outcomes.

### Conclusion, limitations, and further research

Emotional intelligence as the precursor to materialistic inclinations and compulsive buying has received limited attention in academic literature, however, a literature review allows to presume such hypotheses. This study increases our understanding of the impact that emotional intelligence training has on emotional intelligence abilities, materialistic value changes, and changes in compulsive buying tendencies. The contribution of our research may be delineated in two ways. First, this study adds to the limited knowledge on the emotional intelligence training-materialism relationship and the relationship between emotional intelligence training and compulsive buying. Secondly, our study’s unique position within the related literature may be attributed to the experimental research design, which permits the causal claim of the effect of emotional intelligence training on materialism and compulsive buying levels. Previous studies are merely based on the correlational research design when addressing emotional intelligence effect on materialism or related behavioural tendencies (Maddi et al., 2013; Park and Dhandra, 2017; Tariq et al., 2021; Rūtelionė et al., 2022).

A number of limitations must be acknowledged. The first limitation denotes the self-reported measures used to assess study constructs. Though we used scales that are well-established in the literature, such sensitive topics as emotional intelligence abilities and behavioural addiction may be susceptible to social desirability bias. However, we have taken effort to prevent this problem from happening. For instance, the study participants were reassured that their identities and personal information will be protected by anonymising their sensitive information. Moreover, the leading questions, which might impact the respondents’ answer were carefully reworded. Moreover, as the data was collected *via* an online questionnaire, by giving the respondents the time and the place where they are undisturbed by others, we aimed at minimising the social desirability bias. Consequently, the sample size could be listed as the second limitation of our study. On the other hand, compromising with a higher number of subjects in the experimental group could have adversely affected the learning outcomes of the emotional intelligence training programme, which is highly individual-oriented.

The third limitation is associated with the specifics of the sample of the subjects, namely, university students; thus, the study results could not be generalised for other population groups. Finally, compulsive buying was measured as the self-reported attitudinal variable, preventing actual behaviour claims. The attitude-behaviour-gap is a promising research direction in various domains of the consumer behaviour field (Juvan and Dolnicar, 2014; Shaw et al., 2016). Whilst most often studied within sustainable and ethical consumption topics, the same gap may also be observed in the field of maladaptive consumer behaviour; respondents may not be inclined to openly discuss the phenomenon in fear of social repercussions due to its adverse effect on societal well-being.

Further research could address the above limitations by applying different measures in particular with respect to emotional intelligence, which is defined in our study as the ability-based construct. Future studies may benefit from using the CEIS (Consumer emotional intelligence) scale (Kidwell et al., 2008), which is specifically developed to capture consumer-domain emotional intelligence to better fit within the premises of the consumer behaviour field. Future studies should also examine the effect of emotional intelligence on actual behaviour regarding compulsive buying tendencies. An intriguing research perspective could be to explore the role of emotional intelligence in explaining the gap between attitude and behaviour of consumers in the case of compulsive buying. Literature suggests that individuals scoring high in emotional intelligence may possess certain narcissistic characteristics (Petrides et al., 2011). For example, the recent meta-analysis of Michels and Schulze (2021) revealed that narcissism is positively related to trait emotional intelligence. Since narcissistic persons are prone to positive self-bias, it is plausible to assume that such inclination to self-distortion may provide valuable insights on the attitude-behaviour-gap of high narcissism consumers.

It is also worth shedding light on the hierarchical structure of emotional intelligence in future studies. Thus, another angle could be taken to facilitate an understanding of the interdependence of separate facets of emotional intelligence. Studies could test how those interdependent abilities, such as perception, understanding, and regulation of emotions, impact materialistic values and associated behavioural outcomes. There is also a need to understand why individual effects of the components of emotional intelligence are capable of dampening materialistic values and addictive buying tendencies.

### Practical implications

The research revealed that even short-term emotional intelligence training programmes could effectively reduce consumer materialism and compulsive buying. Short-term interventions, along with elements of psychoeducation, can be used to raise consumer awareness. This finding may be helpful for professionals working with individuals who have difficulty with intensified materialistic values or compulsive purchasing and other associated risks.

The current study involved only young consumers as the experiment subjects. The results suggest that young consumers, in this case university students, may benefit from even short training on how to improve their emotional regulation, perceiving and understanding their emotions. According to Twenge and Kasser's (2013) large-scale representative study, poor economic conditions during childhood have a lagging effect on youth’s increased materialism later in life. More specifically, economic deprivation during childhood predicts higher levels of materialism 10 years later. Such lagging effects suggest that emotional intelligence training should take place at an early age when young consumers are more receptive to the information they receive. The inclusion of the EI development training programme in the study curriculums could also be considered. Government organisations could promote this type of training by supporting the development of apps dedicated to emotional intelligence training and conducting appropriate communication campaigns targeting young consumers. Consumption nowadays gains new forms and is not restricted to object acquisition and possession. Widespread vulnerability to excessive internet consumption induces the need to discuss various public policy measures (Lundahl, 2021). Social media consumption may result in personally harmful addictions leading to internet usage disorders. The preventive capacities of emotional intelligence to dampen materialistic desires and maladaptive buying tendencies may also be exploited when faced with highly stressful, death-related and anxiety-provoking situations such as the COVID pandemic. Since consumers exhibiting higher emotional intelligence demonstrate lower susceptibility to materialism and compulsive buying tendencies, the development of emotional intelligence skills may contribute to more sustainable consumer behaviour, mitigating the vulnerability to materialism, and thus related addictive behavioural consequences.

## Data availability statement

The original contributions presented in the study are included in the article/Supplementary material, further inquiries can be directed to the corresponding author.

## Ethics statement

Ethical review and approval was not required for the study on human participants in accordance with the local legislation and institutional requirements. Written informed consent from the [patients/participants or patients/participants legal guardian/next of kin] was not required to participate in this study in accordance with the national legislation and the institutional requirements.

## Author contributions

RL, DA, SN, AR, BS, and EV: conception or design of the work, drafting the article, and critical revision of the article. AR, DA, RL, BS, and EV: data collection. AR, DA, RL, BS, and SN: data analysis and interpretation. All authors contributed to the article and approved the submitted version.

## Funding

This project has received funding from the Research Council of Lithuania (LMTLT), agreement no. S-MIP-20-12.

## Conflict of interest

The authors declare that the research was conducted in the absence of any commercial or financial relationships that could be construed as a potential conflict of interest.

## Publisher’s note

All claims expressed in this article are solely those of the authors and do not necessarily represent those of their affiliated organizations, or those of the publisher, the editors and the reviewers. Any product that may be evaluated in this article, or claim that may be made by its manufacturer, is not guaranteed or endorsed by the publisher.

## Supplementary material

The Supplementary material for this article can be found online at: https://www.frontiersin.org/articles/10.3389/fpsyg.2022.932395/full#supplementary-material
