# Supplementary material for: Reducing consumer materialism and compulsive buying through emotional intelligence training amongst Lithuanian students
Source: Front Psychol. 2022 Oct 18;13:932395. doi: 10.3389/fpsyg.2022.932395 (PMC9622942; doi:10.3389/fpsyg.2022.932395)
Supplement: Supplementary file 1 [file Data_Sheet_1.pdf]

## Annex 1. Measurements

**Materialism** (Richins, 2004)
*Centrality*
1. I usually buy only the things I need. (r)
2. I try to keep my life simple, as far as possessions are concerned. (r)
3. The things I own aren't all that important to me. (r)
4. I enjoy spending money on things that aren't that practical.
5. Buying things gives me a lot of pleasure.
6. I like a lot of luxury in my life.
7. I put less emphasis on material things than most people I know. (r)

*Happiness*
1. I have all the things I really need to enjoy life. (r)
2. My life would be better if I owned certain things I don't have.
3. I wouldn't be any happier if I owned nicer things. (r)
4. I'd be happier if I could afford to buy more things.
5. It sometimes bothers me quite a bit that I can't afford to buy all the things I'd like.

*Success*
1. I admire people who own expensive homes, cars, and clothes.
2. Some of the most important achievements in life include acquiring material possessions.
3. I don't place much emphasis on the amount of material objects people own as a sign of success. (r)
4. The things I own say a lot about how well I'm doing in life.
5. I like to own things that impress people.
6. I don't pay much attention to the material objects other people own. (r)

**Compulsive buying (Valence et al. 1988)**
1.When I have money, I try to spend part or the whole of them
2. I am often impulsive in my buying behaviour.
3. In my opinion, shopping is a way to overcome the stress of everyday life and relax.
4. I sometimes feel that something inside pushed me to go shopping.
5. There are times when I have a strong urge to buy (clothing, books, etc.)
6. At times, I have felt somewhat guilty after buying a product, because it seemed unreasonable.
7. There are some things I buy that I do not show to anybody for fear of being perceived as irrational in my buying behaviour ("a foolish expense').
8 I often have an unexplainable urge, a sudden and spontaneous desire, to go and buy something in a store.
9. As soon as I enter a shopping centre, I have an irresistible urge to go into a shop to buy something.
10. I am one of those people who often respond to direct mail offers (e.g. books & records).
11. I have often bought a product that I did not need, while knowing that I had very little money left.
12. I am a spendthrift.
13. I have sometimes thought "If I had to do it over again, I would ... "and felt sorry for something I have done or said.

**Emotional intelligence (Schutte et al., 1998)**
*Emotions regulation of others*
1. I know when to speak about my personal problems to others.
4. Other people find it easy to confide in me.
11. I like to share my emotions with others.
13. I arrange events others enjoy.
16. I present myself in a way that makes a good impression on others.
24. I compliment others when they have done something well.

26. When another person tells me about an important event in his or her life, I almost feel as though I have experienced this event myself.
30. I help other people feel better when they are down.

*Emotions regulation of self*

2. When I am faced with obstacles, I remember times I faced similar obstacles and overcame them.
3. I expect that I will do well on most things I try.
10. I expect good things to happen
12. When I experience a positive emotion, I know how to make it last.
14. I seek out activities that make me happy.
21. I have control over my emotions
23. I motivate myself by imagining a good outcome to tasks I take on.
28. When I am faced with a challenge, I give up because I believe I will fail. (r)
31. I use good moods to help myself keep trying in the face of obstacles.

*Appraisal of emotions*

5. I find it hard to understand the non-verbal messages of other people. (r)
9. I am aware of my emotions as I experience them.
15. I am aware of the non-verbal messages I send to others.
18. By looking at their facial expressions, I recognize the emotions people are experiencing.
19. I know why my emotions change.
22. I easily recognize my emotions as I experience them.
25. I am aware of the non-verbal messages other people send.
29. I know what other people are feeling just by looking at them.
32. I can tell how people are feeling by listening to the tone of their voice.
33. It is difficult for me to understand why people feel the way they do. (r)

*Utilisation of emotions*

6. Some of the major events of my life have led me to re-evaluate what is important and not important.
7. When my mood changes, I see new possibilities.
8. Emotions are one of the things that make my life worth living.
17. When I am in a positive mood, solving problems is easy for me.
20. When I am in a positive mood, I am able to come up with new ideas.
27. When I feel a change in emotions, I tend to come up with new ideas.
