# Supplementary material for: Reducing consumer materialism and compulsive buying through emotional intelligence training amongst Lithuanian students
Source: Front Psychol. 2022 Oct 18;13:932395. doi: 10.3389/fpsyg.2022.932395 (PMC9622942; doi:10.3389/fpsyg.2022.932395)
Supplement: Supplementary file 2 [file Data_Sheet_2.pdf]

Annex 2. Emotional Intelligence Development Programme outline

The developed programme consists of three meetings of two hours each between the coach and the group participants. A group is made of 9 participants; two groups were formed (18 participants in total).
Session 1: The aim of this session is to develop the participants' ability to accurately identify their emotions at a particular moment; to help understand their aspirations, motives, and incentives to act; to teach to analyse other people's emotions and to effectively decode nonverbal and verbal cues.

Abilities developed	Activity	Brief description	Methods
	Coach Introduction and getting to know group participants. Quick overview of the EI concept and presentation of the purpose and structure of the EI curriculum, as well as the goals of exercise 1.	Identifying participants' expectations. Discussion on the rules of working in a group: active participation; the right to make mistakes; confidentiality, etc. Detailing training methods, EI level change evaluation criteria, etc.	Discussion
The ability to process, understand and regulate emotional information	Task. *Vocabulary of Emotions*<br>The goal is to expand the vocabulary of emotions. Participants are asked to list as many words as possible that reflect positive emotions. Then, in the next column, participants were asked to write as many words as possible that reflect negative emotions.	Knowing more words that describe emotions helps to better express one's emotions and better recognize the emotions of others; it improves well-being and relationships with other people. After the exercise, the plenitude of such descriptive words is discussed.	Discussion, examples, paired exercises
Ability to describe own and other people's emotions	Task. *Wheel of Feelings*<br>This task is a logical continuation of the previous one. The aim is to help participants expand their vocabulary of emotions and teach them to recognize and describe their feelings more accurately.	Participants are presented with an illustration of a wheel of feelings. They are taught how to use the wheel – both in everyday situations and for a more profound reflection.	Discussion, examples
The ability to recognize and identify the emotions of others.	Task. *Photo Game*<br>The goal is to test the participants' ability to identify emotions in photos.	The task is followed by group discussion. Which emotions were easy to identify, and which ones were trickier? What are the differences between recognizing negative and positive emotions?	Discussion, emotion diaries

The ability to recognize non-verbal cues	Task. *Card Game*<br>The aim is to draw the participants' attention to the essential signs of non-verbal expression of emotions. Participants pull out a card with an emotion written on it. Without showing the card to others, they take turns depicting the written emotion. Group members try to guess the emotion depicted.	The task is followed by group discussion. Which emotions were easy to identify, and which ones were trickier? Evaluation of participants' success in decoding nonverbal and verbal cues.	Discussion, feedback
Development of empathy	Task. *"Martian"*.<br>The aim of the task is to observe the nuances of emotion expression in different people. One of the members of the group is a Martian who copies the emotions shown by other members of the group, i.e. mirroring them.	Discussion on whether they succeeded in reflecting the emotion. What signs help judge a specific emotion? What helps make it more accurate – facial expressions, gestures, etc.	Discussion, examples
Ability to decode non-verbal and verbal cues	Summary of training activities	Group members discuss what new knowledge they have gained and what they have learned during the training. Group members assess each other's emotional / psychological status and single out a participant whose status they understood more easily than others.	Discussion, reflection, and feedback

Homework (mandatory) *"EI self-reflection"*. Optional homework *"Colourful emotions"*.

Session 2: The aim of this session is to develop the participants' ability to use conscious information about one's emotions (especially negative) and to adjust one's emotional reactions to oneself, situations, and other people accordingly.

Abilities developed	Activity	Brief description	Methods used
The ability to understand and describe your own emotions.	Brief reflection on the first meeting. Discussion of the homework "EI self-reflection". Stating the purpose of the second meeting.	Taking notice of how engaged the programme participants are in discussing the homework.	Discussion, emotion diaries, readings feedback

The ability to regulate negative emotions.	Task. “Dialogue with Emotion”<br>The goal is to learn to reduce one’s negative emotions. Each participant is invited to imagine the person sitting in front of them as a negative emotion. They are asked to visualise and create as detailed an image as possible. A dialogue with that image begins.	Discussion on how successful the participants were in influencing a negative emotion with the help of their imagination. What negative emotions did the participants choose? What images were created?	Discussion, reflection, feedback
The ability to regulate negative emotions	Task. “Eraser”<br>The aim of this task is to learn to reduce one’s negative emotions. The participants are asked to remember and vividly imagine an unpleasant situation they were in that they would like to erase. The image is “erased” until it disappears completely. Participants are asked to mentally “draw” something nice to replace the erased images.	Discussion on what negative situations were chosen by the participants and whether they managed to reduce the negative situation with the help of their imagination.	Discussion, reflection, feedback
The ability to be aware of emotions.	Task. “Portrait of Emotions”<br>The aim is to learn to analyse subjectively experienced emotions. The coach names an emotion and the participants need to feel it out, i.e., experience it (rather than act it out). Each participant must focus on how they themselves feel a particular emotion and how another person experiences it (through facial expressions, non-verbal cues, paralinguistics).	During the discussion, the coach inquires whether the participants managed to empathise with the emotion, or they just acted it out, and how participants observed the empathy of others.	Discussion, feedback

The ability to consciously make decisions in emotional situations.	Task “Emotion or Reason”<br>The goal is to learn to “turn on” reason in an emotional situation. The coach asks each participant to remember and imagine a real situation when emotions won over reason, which later caused regret.	When discussing, the coach explains the benefits of this exercise for real life situations: stopping yourself makes it easier to decide whether decisions should be made through one’s emotional or rational side.	Discussion, reflection
The ability to regulate emotions: generate positive emotions.	Task. “Brainstorm of Positive Emotions”<br>The goal is to generate positive emotions in one’s daily life that a person experiences infrequently or insufficiently. Participants are invited to come up with at least 15 ideas to experience them more often.	The discussion emphasises that this is simply a brainstorming exercise: it is not necessary to employ these ideas to increase positive emotions in one’s life.	Discussion, emotion diaries
The ability to apply knowledge about the expression of emotions.	Summary of training activities	Group members discuss what new knowledge they have gained and what they have learned during the session. Group members assess each other’s emotional / psychological status and single out a participant whose state they understood more easily than others.	Discussion, paired exercises

Homework (mandatory) *Determining needs based on emotions.*
Homework (mandatory) *“Vocabulary of Emotions”*

Session 3: The aim of this session is to strengthen the newly formed emotional competencies in the group by modelling various realistic situations.

Abilities developed	Activity	Brief description	Methods used
The ability to understand the connections between needs and emotions.	Brief reflection on the second meeting and identifying the purpose of the third meeting. Discussion of homework.	The aim is for all members of the group to take part in the discussion. Questions are encouraged.	Discussion, reflection

The ability to understand the connections between behaviour and emotions.	Task. “Visualisation of behavioural trends”<br>The aim is to use visualisation exercises to encourage participants to look at how participants react to strong emotions/feelings (e.g., in public speeches).	Discussion on how negative/positive emotions encouraged the participants to behave. What did they want to do? How does the behaviour differ depending on whether the scenario was positive or negative?	Discussion, paired exercises
The ability to regulate emotions.	Task. “The chair of the Wise”<br>The aim is to introduce the participants to the model of three-way thinking. The exercise includes an overview of the model, its graphic concept and the participants’ work “sitting on three chairs”.	After the exercise, the coach conducts a discussion about situations in life, where rational or emotional thinking is used, when instead it would be more effective to act wisely; what good would a more extensive use of wise thinking bring?	Discussion, reflection
The ability to understand the reasons for your impulsive behaviour.	Task. “Hot Buttons”<br>The goal of this task is to figure out personal “hot buttons” and learn to respond appropriately to such personality-activating situations. The exercise involves steps aimed at identifying one’s “hot buttons” and personal reactions to them. Discussion on potential coping strategies and their effectiveness.	After the task, the coach conducts a discussion on how successful the participants were in carrying out the task; what coping strategies seem most appropriate; and whether they have been utilised before. Group reflection.	Discussion, reflection, feedback
The ability to understand the connections between needs, behaviour and emotions.	To strengthen the newly formed emotional competencies in the group by modelling various realistic situations	Situations are proposed by group participants.	Discussion, reflection.
	Concluding remarks.<br>The coach summarises the work of the group.	The aim of the discussion is to involve all participants. What knowledge did they gain? What did they understand? What did they learn? In what personal situations can this knowledge be useful?	Discussion, reflection
